# Genetic Dissection of Trabecular Bone Structure with Mouse Intersubspecific Consomic Strains

**DOI:** 10.1534/g3.117.300213

**Published:** 2017-08-29

**Authors:** Taro Kataoka, Masaru Tamura, Akiteru Maeno, Shigeharu Wakana, Toshihiko Shiroishi

**Affiliations:** * Mammalian Genetics Laboratory, Genetic Strains Research Center, National Institute of Genetics, Mishima, Shizuoka 411-8540, Japan; † Department of Genetics, The Graduate University for Advanced Studies (SOKENDAI), Mishima, Shizuoka 411-8540, Japan; ‡ Technology and Development Team for Mouse Phenotype Analysis, RIKEN BioResource Center, Tsukuba, Ibaraki 305-0074, Japan

**Keywords:** trabecular bone structure, C57BL/6J, MSM/Ms, QTL, consomic mouse strains

## Abstract

Trabecular bone structure has an important influence on bone strength, but little is known about its genetic regulation. To elucidate the genetic factor(s) regulating trabecular bone structure, we compared the trabecular bone structures of two genetically remote mouse strains, C57BL/6J and Japanese wild mouse-derived MSM/Ms. Phenotyping by X-ray micro-CT revealed that MSM/Ms has structurally more fragile trabecular bone than C57BL/6J. Toward identification of genetic determinants for the difference in fragility of trabecular bone between the two mouse strains, we employed phenotype screening of consomic mouse strains in which each C57BL/6J chromosome is substituted by its counterpart from MSM/Ms. The results showed that many chromosomes affect trabecular bone structure, and that the consomic strain B6-Chr15^MSM^, carrying MSM/Ms-derived chromosome 15 (Chr15), has the lowest values for the parameters BV/TV, Tb.N, and Conn.D, and the highest values for the parameters Tb.Sp and SMI. Subsequent phenotyping of subconsomic strains for Chr15 mapped four novel trabecular bone structure-related QTL (*Tbsq1-4*) on mouse Chr15. These results collectively indicate that genetic regulation of trabecular bone structure is highly complex, and that even in the single Chr15, the combined action of the four *Tbsq*s controls the fragility of trabecular bone. Given that *Tbsq4* is syntenic to human Chr 12q12-13.3, where several bone-related SNPs are assigned, further study of *Tbsq4* should facilitate our understanding of the genetic regulation of bone formation in humans.

Bone is an important tissue, which not only supports the body but also has the function of storing mineral salts such as calcium and phosphorus. Homeostasis of bone tissue is maintained by bone remodeling. An excess of bone resorption in imbalanced bone remodeling manifests as reduced bone mineral density (BMD), and microstructural deterioration of trabecular bone eventually causes bone diseases such as osteoporosis (Feng and McDonald 2011). BMD and trabecular bone structure are important factors for determining bone strength (Nazarian *et al.* 2008). Defects of these factors increase risk of fracture and affect quality of life. Thus far, many genome-wide association studies (GWAS) of BMD have been performed using dual-energy X-ray absorption scanning, because this method is used as the clinical standard for diagnosing osteoporosis, and it affords a high-throughput assay for BMD. Through this approach, numerous single-nucleotide polymorphisms (SNPs) associated with BMD have been reported in humans, as reviewed recently (Richards *et al.* 2012).

In model animals, a number of BMD-related quantitative trait loci (QTL) have been found by genetic crosses of laboratory mouse strains, indicating that BMD is a typical complex trait and controlled by many genes (Ackert‐Bicknell *et al.* 2010). The majority of these QTL are found in mouse syntenic regions of human BMD-related loci detected by GWAS (Cho *et al.* 2009; Rivadeneira *et al.* 2009; Xiong *et al.* 2009; Ackert‐Bicknell *et al.* 2010; Zhang *et al.* 2010). These observations suggest that the mouse is a good model system to find the genetic factor(s) contributing to skeletal fragility and homeostasis of bone tissue.

In contrast to BMD, information about genetic factors and QTL that affect trabecular bone structure is severely limited. To analyze trabecular bone structure, X-ray micro computed tomography (micro-CT) analysis is essential. Image data obtained by this method provide indispensable information about trabecular bone structure, such as trabecular bone volume fraction (BV/TV), trabecular thickness (Tb.Th), trabecular number (Tb.N), trabecular separation (Tb.Sp), connectivity density (Conn.D), and structure model index (SMI). Of these, BV/TV is the most important parameter, because the level of BV/TV is positively correlated with trabecular bone strength and stiffness (Nazarian *et al.* 2008). In humans, osteoporotic trabecular shows less connectivity and thinner rod-like structures than normal trabecular, indicating that the value of Conn.D is positively correlated, and the value of SMI is negatively correlated, with trabecular bone strength (Brandi 2009). A drawback of this method is that it is not suitable for high-throughput assay, particularly for humans. Therefore, it is challenging to find the genetic factors responsible for trabecular bone structure in humans. On the other hand, X-ray micro-CT analysis has been successfully applied in mouse. For example, it has been reported that age-related changes in trabecular and cortical bone structures in male mice are similar to those in humans (Halloran *et al.* 2002). Moreover, several QTL that affect trabecular bone structure have been found by genetic crosses of laboratory strains (Bouxsein *et al.* 2004; Bower *et al.* 2006; Beamer *et al.* 2012). However, in these genetic studies, standard laboratory mouse strains, namely C57BL/6J (hereafter abbreviated as B6) and C3H/HeJ (C3H), whose genomes are mainly derived from the single subspecies *Mus musculus domesticus*, were used, and our knowledge of genetic factors and QTL that confer phenotypic difference in trabecular bone structure remains limited.

We previously reported B6-MSM consomic mouse strains (B6-ChrN^MSM^), in which each chromosome of the chromosome host strain B6 is replaced by its counterpart from the chromosome donor strain MSM/Ms (hereafter abbreviated as MSM), an inbred strain established from the Japanese wild mouse *M. m. molossinus* (Moriwaki *et al.* 2009). As a consequence of high-degree genome divergence from B6, MSM appeared to have unique complex traits that had never been observed in the standard laboratory strains (Yonekawa *et al.* 1980; Moriwaki 1994; Yonekawa 1994; Moriwaki *et al.* 1999; Takada *et al.* 2008). Moreover, the whole genome sequence of MSM was determined, and >10 million SNPs between MSM and B6 have been identified thus far (Takada *et al.* 2013). Information about the MSM genome and the SNPs for B6 is now freely available on a National Institute of Genetics (NIG) Mouse Genome Database named NIG_MoG (http://molossinus.lab.nig.ac.jp/msmdb/index.jsp) (Takada *et al.* 2015). Taking advantage of these developments, the consomic strains B6-ChrN^MSM^ have been used for genetic studies of a variety of complex traits, elucidating phenotypic effects of individual chromosomes (Takada *et al.* 2008; Takahashi *et al.* 2008a,b, 2010; Nishi *et al.* 2010).

In this study, capitalizing on the unique genetic status of MSM, we first used X-ray micro-CT to investigate the trabecular bone structure of MSM, focusing on the parameters, BV/TV, Tb.N, Conn.D, Tb.Sp, SMI, and Tb.Th, in comparison with those of B6. We found significant strain differences, with MSM having lower values of BV/TV, Tb.N, and Conn.D, and higher values of Tb.Sp and SMI, than B6. Subsequently, we carried out a genetic dissection of the phenotypic effects of individual chromosomes with the full set of B6-ChrN^MSM^ mouse strains. The results revealed that trabecular bone structure is indeed a highly polygenic trait, with many individual chromosomes each having a significant phenotypic effect. Notably, substantial epistasis was found among the individual chromosomes, because the sum of the individual effects often far exceeded the difference between the two parental strains B6 and MSM.

Next, we addressed the phenotypic effects within a single chromosome focusing on chromosome 15 (Chr15), because consomic strain B6-Chr15^MSM^, carrying MSM-derived Chr15, had the lowest values of BV/TV, Tb.N, and Conn.D, and the highest Tb.Sp and SMI, among the full panel of consomic strains, which were almost the same measurement values as MSM. To genetically dissect the phenotypic effects of Chr15, we generated subconsomic strains, in which only a part of the Chr15 is derived from MSM, whereas the rest of Chr15 and all other chromosomes originate from B6. X-ray micro-CT measurement of the trabecular bone structure of these subconsomic strains revealed that multiple genes control the phenotypic effects on Chr15. Finally, we found four novel QTL that affect trabecular bone formation and bone strength.

## Materials and Methods

### Animals

The Animal Care and Use Committee of the NIG approved all of the animal experiments. Development of a full set of consomic strains was reported previously (Takada *et al.* 2008). Briefly, each consomic strain has the B6 genome, except for one chromosome that is replaced by the corresponding chromosome of MSM. The full set of consomic strains, denoted the consomic panel, was established in collaboration between NIG and the Tokyo Metropolitan Institute of Medical Science, and is available from NIG and RIKEN BioResource Center. B6 was purchased from CLEA Japan and maintained at NIG. According to the consomic nomenclature, each strain was named B6-ChrN^MSM^, where N is the number of the chromosome transferred from MSM. All animals were maintained under a 12-hr light/dark cycle (light period, 06:00–18:00; dark period, 18:00–06:00) in a temperature- (23 ± 2°) and humidity-controlled (50 ± 10%) room in a specific pathogen-free area. All mice were weaned after 4 wk of age and housed individually in standard plastic cages on wood chips, and fed a standard diet, CE-2 (CLEA Japan).

### Construction of subconsomic strains

Subconsomic strains possessing subdivided MSM-derived Chr15 were generated by crossing B6 and B6-Chr15^MSM^ (hereafter abbreviated as C15). The F_1_ hybrid mice of B6 and C15 were then backcrossed to B6, and the resultant progeny were genotyped for SNP marker loci; heterozygous mice with an appropriate recombinant breakpoint were intercrossed to obtain homozygotes of the recombinant Chr15 on the B6 genetic background. Established subconsomic strains that harbor various lengths of MSM-derived fragments of Chr15 were named C15_X (hereafter referred to as Sub-X), and maintained as homozygous lines. All subconsomic strains generated in this study are available from the Genetic Strains Research Center at NIG.

### Genotyping

Genotyping of mice was carried out using the Mass ARRAY system (SEQUENOM) according to the manufacturer’s instructions. The DNA markers used to assign detailed recombinant breakpoints in Chr15 between B6 and MSM in the subconsomic strains are listed in the Supplemental Material, Table S1. For determining fine borders of B6 and MSM chromosomal fragments in the subconsomic strains, we designed primer sets to detect size differences in PCR-amplified products that resulted from structural variation such as indels between B6 and MSM genomes (Figure S1).

### X-ray micro-CT analysis

All mice were killed at 6 or 10 wk of age. Bone samples were dissected and fixed in 10% formalin in PBS(-) for 24 hr and then transferred to PBS(-). Bone structure in the metaphysis of the proximal tibia was scanned by micro-CT. Analyses of BV/TV, Tb.N, Conn.D, Tb.Sp, SMI, and Tb.Th were conducted using TRI/3D-BON software (RATOC System Engineering). Bone samples of 6-wk-old B6, C15, and subconsomic mice were scanned using a ScanXmate-L090 micro-CT machine (Comscan Tecno). The image size was set at 1024 × 1024 pixels. Scans were performed using the following parameters: tube voltage peak of 75 kVp, tube current of 52 μA, 360° rotation angle, and 1200 projections. The region of interest (ROI) was 2 mm width from 0.35 mm below the growth plate. Bone samples of 10-wk-old mice were scanned with a ScanXmate-E090S micro-CT scanner (Comscan Tecno). The image size was set at 992 × 992 pixels. Scans were performed using the following parameters: tube voltage peak of 60 kVp, tube current of 130 μA, 360° rotation angle, and 600 projections. The ROI of all mouse strains except for MSM was 1 mm width from 0.36 mm below the growth plate; that of MSM was 0.5 mm width from 0.25 mm below the growth plate, owing to the difference in bone size between MSM and other strains. In all bone imaging experiments, BMD calibration of the micro-CT scanner was carried out every day with a phantom standard provided by the manufacturer. Micro-CT parameters that we used in this study are defined as follows (Bouxsein *et al.* 2010). BV/TV is ratio of the segmented trabecular bone volume to the total volume of the region of interest. Tb.N is a measure of the average number of trabecular per unit length. Conn.D is a measure of the degree of connectivity of trabeculae normalized by total volume of the interest. Tb.Sp is the mean distance between trabeculae. SMI is an indicator of the shape of trabeculae: it is close to 0 if the trabecular network is mainly composed of parallel plates, and near three if cylindrical rods dominate. Tb.Th is the mean thickness of trabeculae.

### Statistical analysis

All data are expressed as mean ± SE. For phenotype screening of the consomic strains at 10 wk of age, all consomic strains were compared with B6 as control, and in statistical analysis Dunnett’s test was performed using EZR (Kanda 2013). Significance was declared when *P* < 0.05. All relationships between two traits were assessed by Spearman’s rank correlation coefficient. Spearman’s rho (*r*) values and their significance were calculated using EZR. Significance was declared when *P* < 0.05. In comparisons of subconsomic strains, a Student’s *t*-test was performed with Welch’s correction. Significance was declared when *P* < 0.005 (*P* of 0.05/10 multiple comparisons).

### Data availability

The B6 strain is commercially available from CLEA Japan. MSM, and all consomic strains and subconsomic strains are available upon request. The DNA markers we used to assign detailed recombinant breakpoints in Chr15 between B6 and MSM in the subconsomic strains are listed in Table S1. File S1 and Table S2 contain detailed micro-CT data for consomic strains. File S2 and Table S3 contain detailed micro-CT data for subconsomic strains. Phenotype data for physiological parameters, body weight, and body length are available from the NIG phenotype database (http://molossinus.lab.nig.ac.jp/phenotype/index.html). The NIG Mouse Genome Database NIG-MoG (http://molossinus.lab.nig.ac.jp/msmdb/index.jsp) was used to determine the SNP information between B6 and MSM for each candidate gene.

## Results

### Phenotype screening of trabecular bone structure for the B6-MSM consomic panel

**Figure 1:**
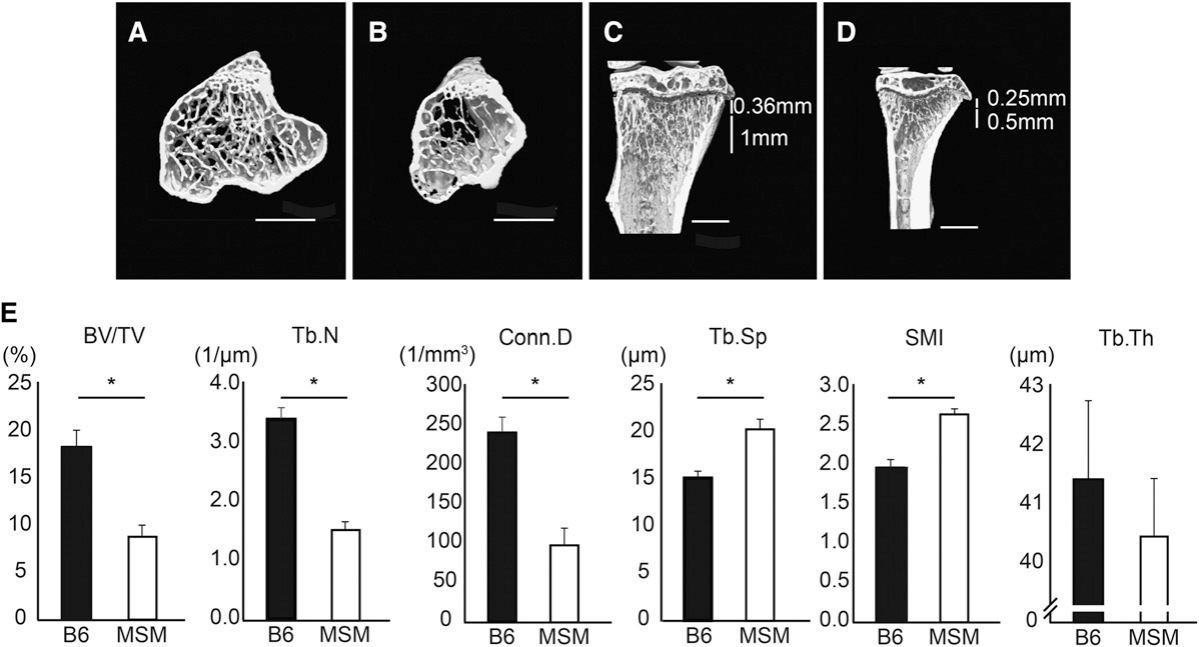
Bone morphology of B6 and MSM at 10 wk of age. (A–D) Representative micro-CT images of the proximal region of tibia of B6 (A and C) and MSM (B and D) mice at 10 wk of age. (A and B) Axial cross-section images; (C and D) sagittal cross-section images. Each ROI is 1 mm in width from 0.36 mm below the growth plate in B6 (C), and 0.5 mm in width from 0.25 mm below the growth plate in MSM (D). Bar, 1 mm. (E) Measurement values of the six parameters, BV/TV, Tb.N, Conn.D, Tb.Sp, SMI, and Tb.Th, of B6 and MSM. Student’s *t*-test with Welch’s correction was performed for statistical analysis. Significance is declared when *P < 0.01.

We obtained X-ray micro-CT images for the proximal metaphyseal region of the tibia of B6 and MSM mice at 10 wk of age, and measured six parameters: BV/TV, Tb.N, Conn.D, Tb.Sp, SMI, and Tb.Th. We compared the measurement values of MSM with those of B6 (Figure 1 and Table S2), and found that the values of BV/TV, Tb.N, and Conn.D of MSM were significantly lower than those of B6, whereas the values of Tb.Sp and SMI of MSM were significantly higher than those of B6. Although a statistically significant difference in the values between B6 and MSM was not observed for Tb.Th, MSM tended to have a lower Tb.Th value than B6 (File S3).

**Figure 2:**
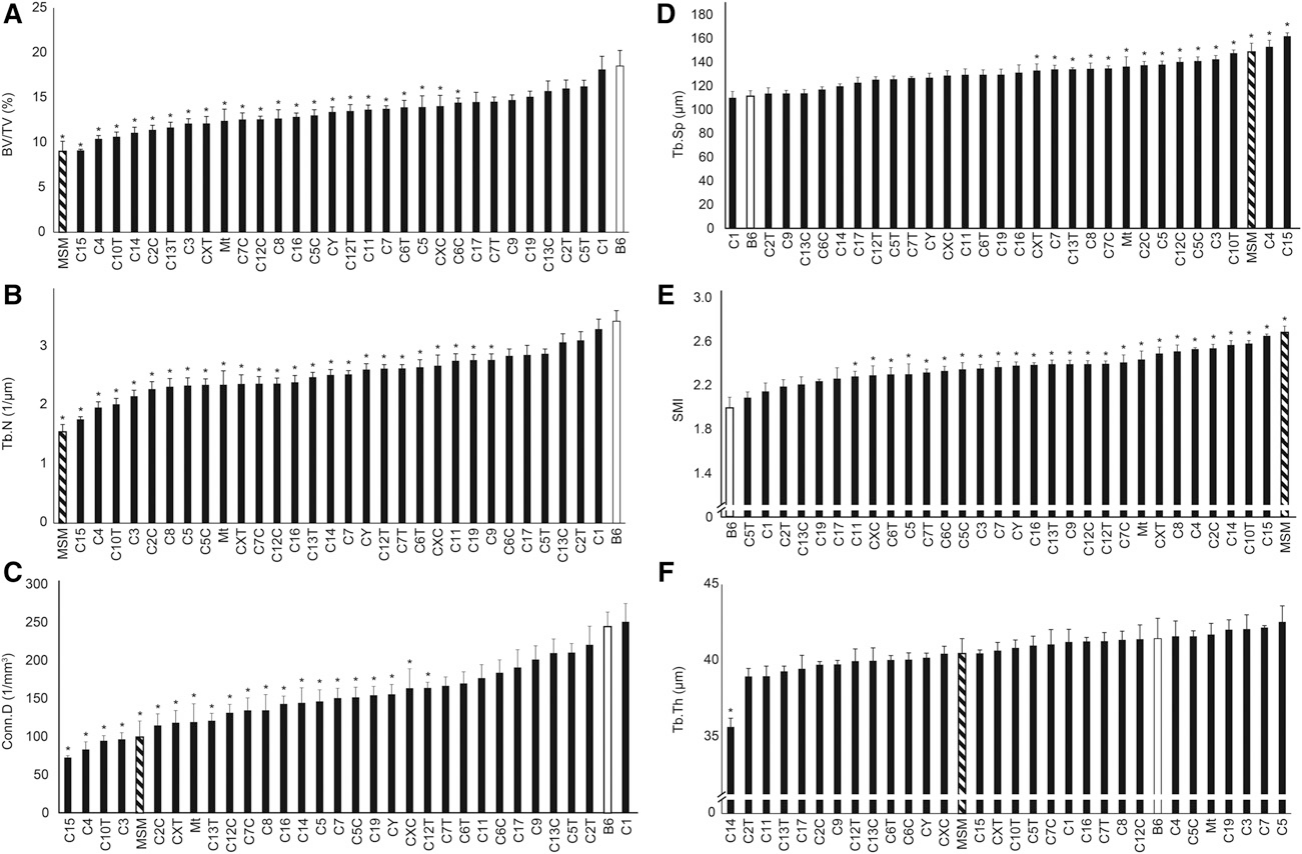
Screening of trabecular bone features among B6, MSM, and the consomic strains. B6, MSM and the consomic strains are aligned in ascending order of each measurement value for the micro-CT results. All measurement values of the parameters BV/TV (A), Tb.N (B), Conn.D (C), Tb.Sp (D), SMI (E), and Tb.Th (F) were obtained from 10-wk-old males. The consomic strain B6-ChrN^MSM^ is abbreviated as CN, where N is the number of the chromosome transferred from MSM. CNC and CNT (*e.g.*, C13C and C13T) denote consomic strains that harbor the centromeric and telomeric half of MSM-derived chromosomes. Y and Mt denote consomic strains that harbor the Y chromosome and mitochondrial genome of MSM, respectively. The measurement values of each consomic strain were compared with those of B6. Dunnett’s test was performed for statistical analysis. Significance is declared when *P < 0.05 (*vs.* control B6).

Next, we obtained micro-CT images at the proximal metaphyseal region of the tibia of the full set of the B6-ChrN^MSM^ consomic panel, and assessed the same six parameters. The results showed large variation in the measurement values of the parameters among the consomic strains (Table S2). We aligned all 26 consomic strains as well as the parental strains, B6 and MSM, in ascending order of the measurement values (Figure 2). With regard to BV/TV and Tb.N, MSM showed the lowest values, and those of all the consomic strains were distributed within the range between MSM and B6 (Figure 2, A and B). A similar strain distribution was observed for Conn.D (Figure 2C), although four consomic strains showed lower values than MSM. Interestingly, most of the consomic strains, including those with the Y chromosome and mitochondrial genome of MSM, showed significantly lower values for BV/TV, Tb.N, and Conn.D than those of B6. Moreover, an inverse strain distribution was observed for the values of Tb.Sp and SMI (Figure 2, D and E). B6 and MSM strains showed extremely low and high values, respectively, and almost all consomic strains were distributed between these parental strains. By contrast, with regard to Tb.Th, there was no statistically significant difference between the parental strains, and the values for many consomic strains exceeded the range between MSM and B6 strains (Figure 2F). In particular, consomic strain C14, which harbors Chr14 of MSM, showed a significantly lower value than B6.

Among all consomic strains, C15, which has MSM-derived Chr15, showed the lowest values of BV/TV, Tb.N, and Conn.D, and the highest values of Tb.Sp and SMI; their values were almost the same as those of MSM (Figure 2). These results implied that mouse Chr15 contains QTL with strong effects on trabecular bone structure, and that Chr15 of MSM tends to decrease BV/TV, Tb.N, and Conn.D, and to increase Tb.Sp and SMI. Notably, C15 has almost the same body size and body weight as B6 (http://molossinus.lab.nig.ac.jp/phenotype/index.html) (Takada *et al.* 2008), suggesting that the trabecular phenotype of C15 is not attributable to secondary effects of the shorter body length and lower body weight of MSM mice.

**Figure 3:**
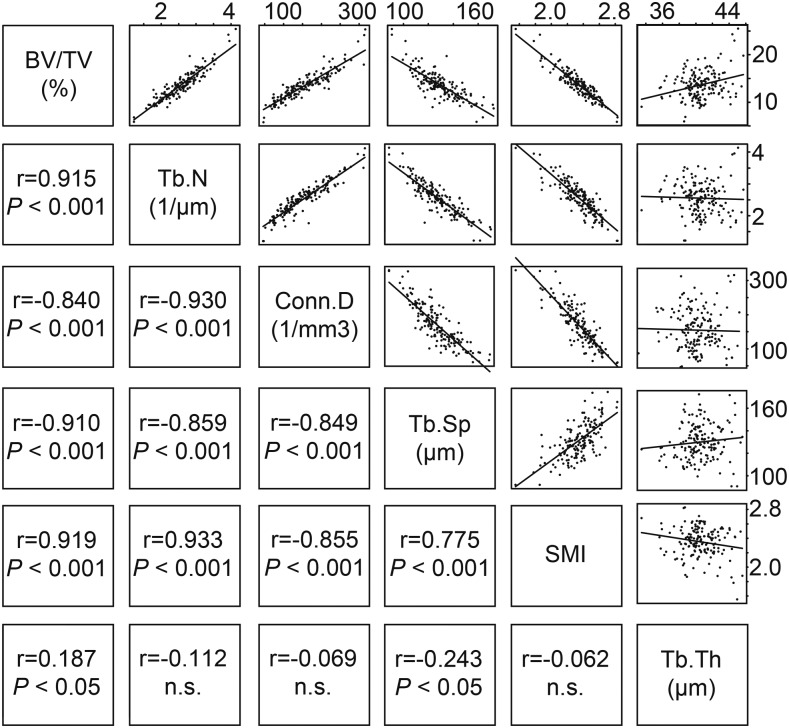
Correlation among six trabecular bone traits in the consomic strains. Correlations between all pairs of the six parameters were examined using data for all individuals in the consomic panel. The cells above and to the right of the name of each parameter display scatter plots for each pair of parameters. Those below and to the left show the corresponding Spearman’s rank correlation coefficients with *P*-values. Significance is declared when *P* < 0.05, and n.s. means not significant.

In the B6-ChrN^MSM^ consomic strains, the ascending orders for BV/TV, Tb.N, and Conn.D were very similar, implying that these parameters correlate with each other. To confirm this correlation, and to establish which parameters are associated with BV/TV and which is the most important parameter for determining the fragility and stiffness of trabecular bone, we investigated correlations for all pairs of BV/TV, Tb.N, Conn.D, Tb.Sp, SMI, and Tb.Th, using the measurement values of all individual samples of the consomic panel. We assessed the correlation coefficients and *P*-values among them (Figure 3). A very strong positive correlation was observed between all pairs of BV/TV, Tb.N, and Conn.D. We also found a strong positive correlation between Tb.Sp and SMI, and these two were negatively correlated with BV/TV, Tb.N, and Conn.D. Between any pair of these five parameters, the absolute *r*-value was >0.77. By contrast, Tb.Th was correlated with none of the other parameters (the absolute *r*-value was <0.25).

### Genetic dissection of trabecular bone structure with C15-derived subconsomic strains

**Figure 4:**
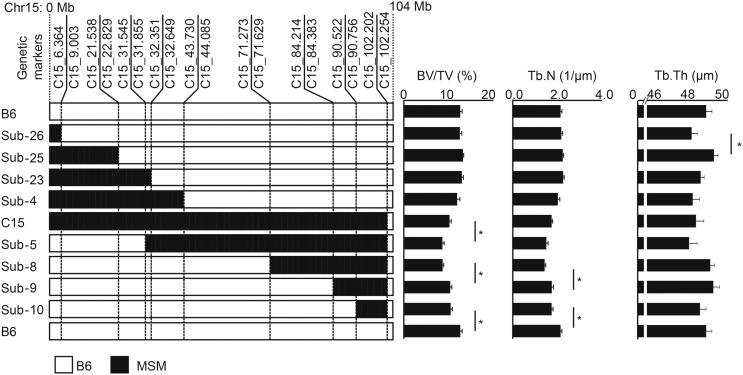
Measurement values of three micro-CT parameters of the subconsomic strains. In the left panel, B6, C15, and the all subconsomic strains are aligned such that two neighboring strains have the minimum difference in the C15 (MSM)-derived chromosomal fragments. The recombination breakpoints that define the borders between B6 and MSM chromosomal fragments in the subconsomic strains are indicated by genetic markers (*e.g.*, C15_6.364 and C15_102.254) at the top of the strain alignment. The genomic information about the DNA markers used for determining the recombinant breakpoints is summarized in Table S1. To the right, the values of BV/TV, Tb.N, and Tb.Th are shown. Significance is declared when *P < 0.005 (0.05/10 comparisons).

To investigate whether a single major gene is responsible for the C15 phenotype or multiple genes confer the phenotype, we generated subconsomic strains that harbor various fragments of MSM-derived Chr15. In total, we successfully established eight subconsomic strains (Figure 4), which were fully fertile and had no reproductive deficiency (data not shown). We obtained X-ray micro-CT images at the proximal metaphyseal region of the all subconsomic strains and measured three parameters, BV/TV, Tb.N and Tb.Th, to narrow down the genetic region(s) responsible for trabecular bone structure. Because the difference in BV/TV and Tb.N between B6 and C15 was observed at as early as 6 wk of age, we carried out phenotyping of these subconsomic strains at 6 wk of age (Figure 4).

**Figure 5:**
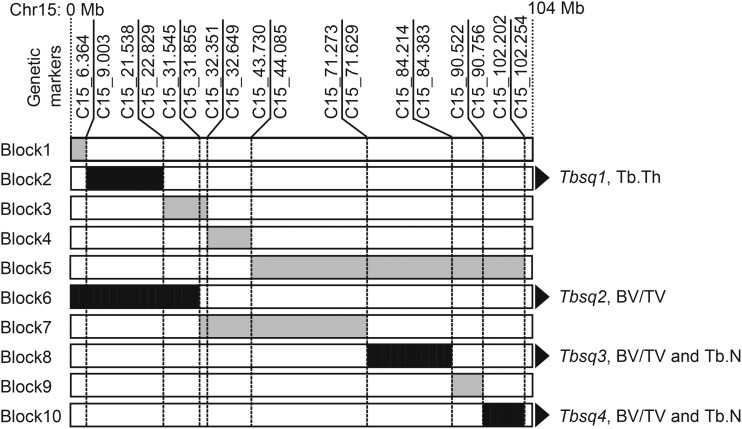
Chromosomal blocks and four trabecular bone structure-related QTL (*Tbsq1* to *4*). Ten chromosomal blocks (gray and black segments) are defined by the difference in chromosomal composition between the neighboring two strains. Among the 10, four black segments contain QTL, named *Tbsq1* to *4*. The parameters affected by these QTL are shown at the right side of the blocks.

To assign chromosomal fragments that contain QTL responsible for the differences in the values of BV/TV, Tb.N, and Tb.Th between B6 and C15, we aligned the eight subconsomic strains as well as B6 and C15 in order to minimize the difference in the length of the MSM-derived chromosomal fragment between two neighboring strains (Figure 4). Comparison of the measurement values between each pair of neighboring strains showed a statistically significant difference in four of the 10 pairs of strains. This result indicated that four QTL affecting trabecular bone structure exist in mouse Chr15. Each pair of two neighboring strains defined 10 separate chromosomal fragments. We numbered these chromosomal fragments from Block1 to Block10 (Figure 5, gray and black chromosomal segments). The four QTL are contained in Block2, Block6, Block8, and Block10, which are defined by comparison between two subconsomic strains, namely Sub-26 and Sub-25, C15 and Sub-5, Sub-8 and Sub-9, and Sub-10, and B6, respectively (Figure 5, black chromosomal segments). We named these QTL trabecular bone structure quantitative locus 1–4 (*Tbsq1-4*).

*Tbsq1* resides in Block2, which is located at the centromeric region of Chr15, and the MSM allele at this locus increases Tb.Th. *Tbsq2* in Block6 affects BV/TV, and the MSM allele at this locus increases the value of BV/TV. Block6 includes Block2 that harbors *Tbsq1*, but no significant difference in Tb.Th was observed between Sub-5 and C15. *Tbsq3* in Block8 affects both BV/TV and Tb.N, and the MSM allele at this locus decreases the value of the above two parameters. *Tbsq4* in Block10, located at the telomeric region of Chr15, affects both BV/TV and Tb.N, and the MSM allele at this locus significantly decreases the value of the above two parameters.

## Discussion

In this study, mouse intersubspecific genome differences between the standard laboratory strain B6 and the Japanese wild mouse-derived strain MSM allowed us to dissect genetic determinants that regulate trabecular bone structure. As a result, we found that trabecular bone structure regulation is extensively polygenic in mouse. Phenotyping with the B6-ChrN^MSM^ consomic strains revealed pervasive QTL that affect the parameters BV/TV, Tb.N, Conn.D, Tb.Sp, and SMI on the mouse genome. BV/TV is known to be the most important parameter for determining the fragility of trabecular bone (Nazarian *et al.* 2008). The present study revealed that roughly two thirds of the chromosomes or chromosomal regions harbored QTL affecting BV/TV, indicating that a large portion of mouse chromosomes contributes to the physical strength of trabecular bone (Figure 2). Notably, we also showed unequivocally that the Y chromosome and the mitochondrial genome possess QTL affecting trabecular bone strength, which has not been reported before.

Recently, QTL affecting trabecular bone structure in mice were reported based on the genetic cross of B6 and another laboratory strain, C3H (Beamer *et al.* 2012). Using nested congenic mouse strains, at least 10 QTL were assigned at the mid-distal region of Chr4 that affect the bone-related traits measured by peripheral quantitative CT and/or micro-CT. Our study also showed that the consomic strain C4, which harbors MSM-derived Chr4, had the second-lowest values for BV/TV, Tb.N and Conn.D, following consomic C15, and this result indicated that Chr4 has the second-largest phenotypic effect on trabecular bone strength. It is possible that the causative genome variation(s) responsible for the reduced trabecular bone strength of C3H originated from the Japanese subspecies *M. m. molossinus*.

As a striking feature of the gene regulation involved in trabecular bone structure, we found extensive nonadditive phenotypic effects on trabecular bone structure. With regard to the measurement values of BV/TV and Tb.N, summation of the phenotypic effects of individual chromosomes far exceeded the difference between the two parental strains, B6 and MSM. For example, summation of the phenotypic effects of 22 strains that showed a statistically significant difference in the BV/TV value from that of B6 yielded 1390% of the parental difference. A similar result was also found for Tb.N, where the sum of the phenotypic effects was 1291% of the parental difference. Such strong epistatic effects have often been reported in phenotyping of mouse consomic strains for many other complex traits (Shao *et al.* 2008; Takada *et al.* 2008). With respect to Tb.Th, only subconsomic strain C14 demonstrated significantly lower trabecular thickness than B6. This suggests that disruption of an epistatic gene interaction between the MSM allele in Chr14 and B6 gene(s) in other chromosome(s) gives rise to the phenotype.

In this study, we investigated the correlation coefficients among six parameters, all of which were related to trabecular bone structure. We observed strong positive correlations in every pair of BV/TV, Tb.N, and Conn.D, and between Tb.Sp and SMI. The former three parameters showed negative correlations with the latter two. Considering these positive and negative correlations among the parameters, a lower value of BV/TV indicates not only fewer trabecular bones, but is also associated with morphological features such as rod-shaped trabecular bones and disconnected trabecular bones. The observed correlations between the five parameters suggest that they are regulated by common genetic factor(s). On the other hand, the parameter Tb.Th did not correlate with any of the other parameters. Therefore, the genetic factors contributing to Tb.Th are independent of those contributing to the other parameters.

It has been reported that values of BV/TV, Tb.N, and Conn.D in mice peak at ∼6 wk of age and gradually decrease with age, whereas, conversely, Tb.Sp and SMI increase with age. By contrast, Tb.Th does not change significantly with age. The decrease of BV/TV, Tb.N, and Conn.D, and the increase of Tb.Sp and SMI, occur linearly with age, and the values of the parameters at the early phase (6–10 wk of age) are important for predicting trabecular strength in the later life of mice (Halloran *et al.* 2002). We inferred that the genetic factors contributing to BV/TV, Tb.N, and Conn.D could be involved in the formation of trabecular bones at early stages of the life span, rather than in the regulation of homeostasis of bone remodeling.

This study showed that Chr15 has the strongest genetic influence on the trabecular bone structure of mice, and that it contains four novel trabecular bone structure-related QTL (*Tbsq1-4*). The MSM alleles at two of these loci, *Tbsq3* and *4*, decrease the BV/TV, reflecting the phenotype of the original consomic strain C15. The MSM allele at *Tbsq2* acts in the opposite way to increase the BV/TV. The MSM allele at *Tbsq1* increases the Tb.Th, although the original consomic strain C15 does not show a significant difference in Tb.Th compared with B6. We searched public databases and previous reports for candidate genes for *Tbsq1-4*. As a result, we identified a total of 20 candidate genes in the genomic regions encompassing the four QTL (Table 1). Among these, 12 have nonsynonymous SNPs between the B6 and MSM genomes, and nine have been reported to be involved in bone formation or homeostasis by *in vivo* assays. Although these nine genes are good candidates for the QTL, other genes that have only synonymous SNPs or no SNPs in their coding sequences cannot be excluded from the list of candidate genes. If these genes had SNPs in their *cis*-regulatory elements, such as the promoter and enhancer, gene expression could be altered, and the SNPs and other structure variants could eventually cause the phenotype. The Block10 region that contains *Tbsq4* is syntenic to human Chr12q12-13.3, where several bone-related SNPs have been assigned from GWAS (Bezerra *et al.* 2008; Gentil *et al.* 2007, 2009; Kim *et al.* 2007; Pérez *et al.* 2008; Dundar *et al.* 2009; Mencej-Bedrač *et al.* 2009; Pluskiewicz *et al.* 2009; Timpson *et al.* 2009; Zmuda *et al.* 2009). Identification of the causative gene(s) for *Tbsq4* should facilitate our understanding of the genetic regulation of bone structure in humans. In any case, further studies are needed to reveal the causative genes for *Tbsq1-4*.

**Table 1:** Proposed candidate genes for four QTL in mouse Chr15: physical region, candidate genes, biological effects, and SNP information between B6 and MSM

QTL (Block)	Genetic Region (Mb)	Gene Symbol	Gene Function in Bone	Information About SNPs and Indels*^a^*
*Tbsq1* (Block2)	6.64–21.75	*Rictor*	Skeletal growth and bone anabolism (Chen *et al.* 2015).	7/2/0/0
		*Osmr*	Promotion of bone formation (Walker *et al.* 2010).	6/8/1/1
		*Lifr*	Osteoclast number (Ware *et al.* 1995).	12/6/0/0
		*Cdh6*	Osteoclast maturation (Mbalaviele *et al.* 1998).	12/2/0/0
*Tbsq2* (Block6)	0–32.35	*Ghr*	Bone growth (Sjogren *et al.* 2000).	3/3/0/0
		*Ptger4*	PGE_2_ receptor. Bone formation (Akhter *et al.* 2006).	3/0/0/0
		*Myo10*	Osteoclast bone resorption *in vitro* (McMichael *et al.* 2010).	26/4/0/0
		*Ank*	Ossification (Ho 2000).	7/0/0/0
*Tbsq3* (Block8)	71.63–84.21	*Ptk2*	Osteoblast mechanotransduction *in vitro* (Castillo *et al.* 2012).	6/3/0/0
		*Ly6a*	Age-dependent osteoporosis (Bonyadi *et al.* 2003).	0/0/0/0
		*Recql4*	Osteoprogenitor proliferation (Hoki *et al.* 2003; Yang *et al.* 2006).	3/4/0/0
		*Pdgfb*	Bone metabolism (Xie *et al.* 2014).	0/0/0/0
		*Atf4*	Osteoblast differentiation (Yang *et al.* 2004).	3/0/0/0
		*Mchr1*	Cortical BMD (Bohlooly *et al.* 2004).	1/0/0/0
		*Tob2*	*Rankl* expression and osteoclast differentiation (Ajima *et al.* 2008).	2/1/0/0
		*Scube1*	Early cranial bone formation (Tu *et al.* 2008).	18/6/0/0
*Tbsq4* (Block10)	90.76–102.20	*Vdr*	Bone homeostasis (Yoshizawa *et al.* 1997; Yamamoto *et al.* 2013) and human GWAS (Gentil *et al.* 2007, 2009; Kim *et al.* 2007; Bezerra * et al.* 2008; Pérez *et al.* 2008; Dundar *et al.* 2009; Mencej-Bedrač * et al.* 2009; Pluskiewicz *et al.* 2009).	8/9/0/0
		*Col2a1*	Endochondral ossification (Li *et al.* 1995).	10/1/0/0
		*Wnt10b*	Osteoblast differentiation (Bennett *et al.* 2005) and human GWAS (Zmuda *et al.* 2009).	6/0/0/0
		*Sp7*	Osteoblast differentiation (Nakashima *et al.* 2002) and human GWAS (Timpson *et al.* 2009).	0/0/0/0

a No. of synonymous SNPs/nonsynonymous SNPs/insertions/deletions.

Collectively, the results of this study demonstrate that the mouse genome encodes numerous genetic factors regulating trabecular bone structure. Considering the phenotypic effects of the four QTL identified in Chr15, many other QTL may have modest effects on bone phenotypes. It would be very difficult to detect such QTL using linkage analysis by general outcross experiments, F_1_ intercross, and backcross. Thus, this study has also revealed the marked complexity of genetic architecture that controls trabecular bone structure in mouse, and demonstrated that analysis with consomic and subconsomic strains has considerable power to extract each of numerous QTL, even if its phenotypic effect is modest.

## Supplementary Material

Supplemental material is available online at www.g3journal.org/lookup/suppl/doi:10.1534/g3.117.300213/-/DC1.
