# Supplementary material for: Genetic Dissection of Trabecular Bone Structure with Mouse Intersubspecific Consomic Strains
Source: G3 (Bethesda). 2017 Aug 29;7(10):3449–57. doi: 10.1534/g3.117.300213 (PMC5633393; doi:10.1534/g3.117.300213)
Supplement: Supplementary file 7 [file 3449FileS3.docx]

**Legends for supplementary files**

**File S1**

A list of micro-CT measurement values of individual trabecular samples of the full consomic panel. The values of six micro-CT parameters were measured for the consomic mice at 10 weeks of age.

**File S2**

A list of micro-CT measurement values of the sub-consomic strains. The values of six micro-CT parameters were measured for the sub-consomic mice at six weeks of age.
